# Alpha-synuclein mRNA expression in oligodendrocytes in MSA

**DOI:** 10.1002/glia.22653

**Published:** 2014-03-03

**Authors:** Yasmine T Asi, Julie E Simpson, Paul R Heath, Stephen B Wharton, Andrew J Lees, Tamas Revesz, Henry Houlden, Janice L Holton

**Affiliations:** 1 Queen Square Brain Bank Department of Molecular Neuroscience, UCL Institute of NeurologyLondon, United Kingdom; 2 Sheffield Institute for Translational Neuroscience, University of SheffieldSheffield, United Kingdom; 3 Reta Lila Weston Institute of Neurological Studies, UCL Institute of NeurologyLondon, United Kingdom; 4 Department of Molecular Neuroscience, UCL Institute of NeurologyLondon, United Kingdom

**Keywords:** α-synuclein, multiple system atrophy, oligodendrocytes, glial cytoplasmic inclusions, laser-capture microdissection

## Abstract

Multiple system atrophy (MSA) is a progressive neurodegenerative disease presenting clinically with parkinsonian, cerebellar, and autonomic features. α-Synuclein (αsyn), encoded by the gene *SNCA*, is the main constituent of glial cytoplasmic inclusion (GCI) found in oligodendrocytes in MSA, but the methods of its accumulation have not been established. The aim of this study is to investigate alterations in regional and cellular *SNCA* mRNA expression in MSA as a possible substrate for GCI formation. Quantitative reverse transcription polymerase chain reaction (qPCR) was performed on postmortem brain samples from 15 MSA, 5 IPD, and 5 control cases to investigate regional expression in the frontal and occipital regions, dorsal putamen, pontine base, and cerebellum. For cellular expression analysis, neurons and oligodendrocytes were isolated by laser-capture microdissection from five MSA and five control cases. *SNCA* mRNA expression was not significantly different between the MSA, IPD and control cases in all regions (multilevel model, *P =* 0.14). After adjusting for group effect, the highest expression was found in the occipital cortex while the lowest was in the putamen (multilevel model, *P <* 0.0001). At the cellular level, MSA oligodendrocytes expressed more *SNCA* than control oligodendrocytes and expression in MSA neurons was slightly lower than that in controls, however, these results did not reach statistical significance. We have demonstrated regional variations in *SNCA* expression, which is higher in cortical than subcortical regions. This study is the first to demonstrate *SNCA* mRNA expression by oligodendrocytes in human postmortem tissue using qPCR and, although not statistically significant, could suggest that this may be increased in MSA compared to controls.

## Introduction

Multiple system atrophy (MSA) is a progressive neurodegenerative disease that clinically presents with parkinsonian, cerebellar and autonomic features and pathologically with glial cytoplasmic inclusions (GCIs) found in oligodendrocytes, myelin damage, neuronal loss, and gliosis (Ahmed et al. 2012; Ubhi et al. 2011). The main constituent of GCIs is α-synuclein (αsyn), as such, classifying MSA as part of the α-synucleinopathy group of diseases, which also includes Parkinson's disease (PD) and dementia with Lewy bodies (DLB). MSA is pathologically subtyped into striatonigral (SND), olivopontocerebellar (OPC) or mixed type reflecting the predominance of pathological burden in the respective anatomical structures. However, the basis for this regional vulnerability is poorly understood (Jellinger et al. 2005; Ozawa et al. 2004). The mechanism by which αsyn accumulates in oligodendrocytes as GCIs is as yet unknown but is hypothesized to result from the uptake of αsyn, released from neurons, by oligodendrocytes or by overexpression of *SNCA* mRNA in the disease condition (Reyes et al. 2014; Ubhi et al. 2011). Mutations and multiplications in *SNCA*, the gene encoding αsyn, have been identified in some cases of familial PD and these have occasionally been found to have oligodendroglial inclusions resembling GCIs (Gwinn-Hardy et al. 2000; Kiely et al. 2013; Markopoulou et al. 2008; Obi et al. 2008). Despite this, no mutations in *SNCA* have been associated with MSA (Gwinn-Hardy et al. 2000; Kiely et al. 2013; Markopoulou et al. 2008; Obi et al. 2008). There are limited studies in the literature of *SNCA* expression in MSA. In a small case study, *SNCA* mRNA levels in cortical regions (frontal, temporal, or occipital) of MSA brains did not differ from those in controls using quantitative PCR (qPCR) (Ozawa et al. 2001). Cortical regions are less severely affected by GCI pathology and neuronal loss in MSA and this may explain these results. Based on findings of our study, it may be plausible to suggest that expression differences are more likely to arise in areas more severely affected in MSA such as the pons and cerebellum. However, there are conflicting data with regards to *SNCA* expression in the pons. Jin et al. (2008) observed no difference in *SNCA* copy number and mRNA expression in the pons of MSA and control cases, while down-regulation was reported by Langerveld et al. (2007). Different isoforms of *SNCA* resulting from alternative mRNA splicing were investigated in PD, DLB, and MSA and isoform *SNCA* 98 was found to be up-regulated in the frontal cortex of MSA, while there was no significant difference in the level of *SNCA* 126 (Beyer et al. 2008).

Previous studies focused on understanding *SNCA* mRNA expression in MSA at the regional level and there is little information relating to cellular expression. *In situ* hybridization studies of *SNCA* mRNA expression in PD and control cases have successfully identified expression in neurons but not in oligodendrocytes (Jin et al. 2008; Kingsbury et al. 2004; Miller et al. 2005; Solano et al. 2000). Unravelling the cellular expression profile of *SNCA* in MSA will help to shed light on the possible contribution of overexpression in oligodendrocytes as a mechanism for GCI formation. Therefore, the aim of this study was to investigate the regional and cellular expression profile of *SNCA* in MSA subtypes and controls using qPCR.

## Materials and Methods

### Tissue Collection

Samples were obtained from brains donated to the Queen Square Brain Bank (QSBB) for Neurological Disorders, Department of Molecular Neuroscience, UCL Institute of Neurology, London. The donations were made according to ethically approved protocols, and tissue is stored under a license issued by the Human Tissue Authority (No. 12198). One half of the brain is sliced in the coronal plane, flash frozen, and stored at −80°C on arrival to the QSBB, while the other half is fixed in 10% buffered formalin. Tissue was sampled from the frozen half brain.

### Case Selection

MSA cases were pathologically typed based on published criteria into mixed, SND and OPCA subtypes (Ozawa et al. 2004). Five MSA-SND, five MSA-OPCA, five MSA-mixed subtypes were selected and sex and age matched to five IPD and four normal control cases to study regional αsyn mRNA expression (Table1). For cellular mRNA expression, five MSA-mixed subtype and six neurologically normal controls were used to isolate neurons and oligodendrocytes (Table2).

**Table 1:** Demographics of Regional Expression Cohort

Case	Age	Sex	Diagnosis	MSA pathological type	PMI (hr)
1	56	F	MSA	Mixed	28
2	62	M	MSA	Mixed	20
3	64	F	MSA	Mixed	52
4	66	M	MSA	Mixed	105
5	70	F	MSA	Mixed	65
6	50	F	MSA	SND	4
7	54	F	MSA	SND	47
8	58	F	MSA	SND	11
9	63	M	MSA	SND	27
10	72	M	MSA	SND	50
11	56	M	MSA	OPCA	37
12	60	F	MSA	OPCA	62
13	61	M	MSA	OPCA	ND
14	64	M	MSA	OPCA	35
15	66	F	MSA	OPCA	74
16	61	F	IPD	NA	26
17	63	M	IPD	NA	37
18	65	M	IPD	NA	43
19	67	F	IPD	NA	108
20	70	M	IPD	NA	75
21	57	M	Control	NA	79
22	69	M	Control	NA	ND
23	71	M	Control	NA	39
24	73	F	Control	NA	24

NA: not applicable; ND: not documented; PMI: post-mortem interval.

**Table 2:** Demographics of Cellular Expression Cohort

Case	Age	Sex	Diagnosis	MSA pathological type	PMI (hr)	RIN before LCM	RIN after LCM
1	50	M	MSA	Mixed	30	4.8	2.6
2	70	F	MSA	Mixed	65	5.8	ND
3	56	M	MSA	Mixed	75	4.7	ND
4	64	F	MSA	Mixed	99	4.4	2.8
5	64	M	MSA	Mixed	100	5.1	3.1
6	69	M	Control	NA	ND	4.4	ND
7	82	F	Control	NA	ND	4.8	3.6
8	73	F	Control	NA	24	5.3	2.0
9	85	M	Control	NA	78	4.2	2.2
10	80	F	Control	NA	49	3.9	2.5
11	83	M	Control	NA	63	4.2	2.3

NA: not applicable; ND: not documented; PMI: post-mortem interval.

### Regional Sampling, RNA Extraction, and Reverse Transcription

Frozen tissue (∼100 mg) from the posterior frontal region (cortex and subcortical white matter), occipital region (cortex and subcortical white matter), dorsal putamen, pontine base, and cerebellum (white matter) was collected and homogenised using TissueRuptor (Qiagen, Germany). RNA extraction was carried out using RNeasy Mini Kit (Qiagen, Germany) and cDNA synthesized using SuperScript® VILO™ cDNA Synthesis Kit (Invitrogen, UK) according to the manufacturer's instructions.

### Cellular Sampling, RNA Extraction, and Reverse Transcription

Neurons from the pontine base (∼1,000 cells) and oligodendrocytes from the cerebellar white matter (∼1,000 cells) were isolated in five MSA and six control cases using the PixCell II laser-capture microdissection (LCM) system (Arcturus Engineering, Mountain View, CA). Neurons were identified using toluidine blue stain and oligodendrocytes using a rapid immunohistochemistry (IHC) protocol as previously described (Waller et al. 2012). Briefly, 7-µm frozen section were collected on uncharged, sterile glass slides and warmed to RT for 30 sec. The sections were then fixed in ice-cold acetone for 3 min and immunostained using oligodendrocyte-specific protein (OSP) primary antibody (Abcam, UK, ab7474) and VECTASTAIN Elite ABC kit (Vector labs, Burlingame, CA, PK-6101). Sections were blocked with normal goat serum (2%) for 3 min then incubated in OSP (1:10) for 3 min at RT. After a brief rinse with tris-buffered saline (TBS), sections were incubated in 5% biotinylated secondary antibody for 3 min at RT, then rinsed again with TBS. The avidin–biotin complex solution was then added to the section for 3 min at RT then rinsed off. DAB peroxidase substrate kit (Vector labs, Burlingame, CA, SK-4100) was used to visualize the reaction by incubating section for 3 min at RT. The sections were rinsed with TBS, dehydrated in graded alcohol (70, 95, 100, 100% for 1 min each), cleared in two changes of xylene and allowed to air dry in an air-flow hood for an hour prior to LCM. This rapid IHC protocol was carried out under sterile conditions and using diethylpyrocarbonate (DEPC)-treated water. RNA extraction of LCM samples was carried out using the Arcturus PicoPure RNA isolation kit (Applied Biosystems, UK, KIT0204) and cDNA synthesized using SuperScript® VILO™ cDNA Synthesis Kit (Invitrogen, UK) according to manufacturer's instructions. RNA quality and concentration was then assessed using the Nanodrop and Agilent Bioanalyser 2100. Maintenance of RNA quality was adequate as assessed by the RIN of samples before and after LCM was carried out. Prior to LCM, samples had a mean RIN of 4.7 (range 4.2–5.8) declining to 2.6 (range 2.0–3.1) after LCM and this is in keeping with previous findings (Waller et al. 2012).

### Quantitative PCR

qPCR was performed on Stratagene MX3000p (Agilent technologies, CA) using 50-ng cDNA and Power SYBR Green Master Mix (Applied Biosystems) according to the manufacturer's instructions. All primers were run at the following thermal profile: one cycle at 95°C for 10 min followed by 40 cycles at 95°C for 15 min and 60°C for 1 min. Three reference genes, UCB, TBP, and GAPDH, were determined as appropriate to normalize regional expression data using qBase plus software (Biogazelle, Belgium). Due to limited sample volume, one reference gene (UBC) was used to normalize LCM expression data. Primers for the gene of interest (GOI), *SNCA*, were designed in house while the reference genes were obtained from RTprimerDB (Table3).

**Table 3:** Primers Information

Gene	SNCA	TBP	UBC	GAPDH
Gene name	synuclein, alpha (non A4 component of amyloid precursor)	TATA box binding protein	Ubiquitin C	Glyceraldehyde-3-phosphate dehydrogenase
RefSeq no.	NM_001146055.1	NM_003194	NM_021009	NM_002046
Primer sequence	**F:** CAACAGTGGCTGAGAAGACCA	**F:** TGCACAGGAGCCAAGAGTGAA	**F:**	**F:** GAAATCCCATCACCATCTTCCAGG
	**R:** GCTCCTTCTTCATTCTTGCCCA	**R:** CACATCACAGCTCCCCACCA	ATTTGGGTCGCGGTTCTTG	**R:**
			**R:** TGCCTTGACATTCTCGATGGT	GAGCCCCAGCCTTCTCCATG
Alignment	**F:** base 228 to 249	**F:** base 892 to 913	**F:** base 399 to 418	**F:** base 313 to 337
	**R:** base 369 to 390	**R:** base 1004 to 1024	**R:** base 511 to 532	**R:** base 413 to 433
Amplicon length (bp)	163	132	133	120
Organism	Homo sapiens	Homo sapiens	Homo sapiens	Homo sapiens
Source	In house design	RTPrimerDB (ID: 2630)	RTPrimerDB (ID: 8)	RTPrimerDB (ID: 1108)

### Expression Analysis and Statistics

Standard curves were used to extrapolate expression value of each gene in each region or cell. The GOI was then normalized to the geometric mean of the reference genes as recommended by Vandesompele et al. (2002). A multilevel statistical model was used for regional expression analysis while the Mann–Whitney U test was used to analyze cellular expression results with the significance levels set at *P* < 0.05.

## Results

**Figure 1:**
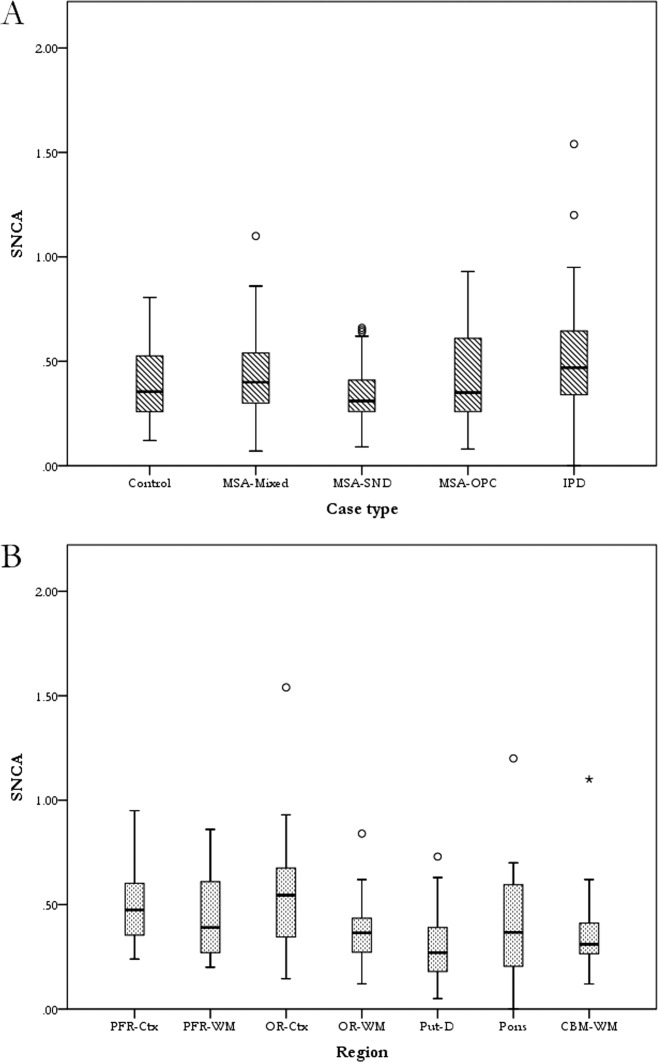
*SNCA* mRNA regional expression (A) Expression between the different cases (adjusted for regions) shows no statistically significant differences between the groups although the lowest level of expression was found in MSA-SND and the highest in IPD cases (*P =* 0.14). (B) After adjusting for case type, a statistically significant difference in *SNCA* expression between regions was found (*P <* 0.0001), with the lowest expression in the dorsal putamen and the highest in the occipital cortex. Multilevel model test with significance level set at *P* = 0.05. The boxplot show median values as the line within the box, the box reflects the interquartile range and the whiskers the range of the values.PFR: posterior frontal region; OR: occipital region; Put-D: dorsal putamen; CBM: cerebellum; Ctx: cortex; WM: white matter; MSA: multiple system atrophy; SND: striatonigral degeneration; OPC: olivopontocerebellar; IPD: idiopathic Parkinson's disease; *SNCA*: α-synuclein.

Regional *SNCA* mRNA expression was examined in the posterior frontal region, occipital region, dorsal putamen, pontine base, and cerebellar white matter of three MSA subtypes (mixed, SND, and OPCA), IPD, and control cases (Fig. 1). *SNCA* expression level was highest in IPD and lowest in MSA-SND, however, data analysis using a multilevel statistical model showed that there was no statistically significant difference between the different groups (multilevel statistical model, *P =* 0.14). After adjusting for group effect, the highest expression was found in the occipital cortex while the lowest was in the putamen. The differences between regions were found to be statistically significant (multilevel statistical model, *P <* 0.0001).

**Figure 2:**
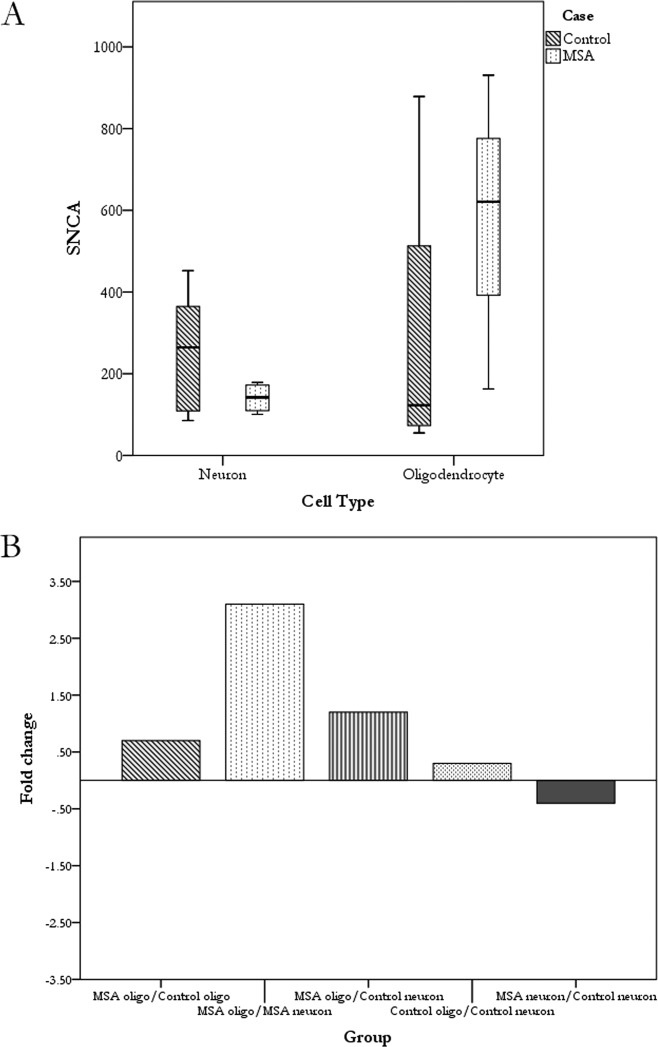
*SNCA* mRNA cellular expression(A) *SNCA* is expressed in neurons and oligodendrocytes of both MSA and control cases. Expression in neurons is greater in controls as compared to MSA (*P =* 0.47), in contrast to oligodendrocytes where expression is greater in MSA (*P =* 0.18) but these results did not reach statistical significance. (B) Bar graph representing fold change of expression values between the different cases and cell types. No statistically significant differences were found although there was a trend toward increased expression in MSA oligodendrocytes when compared to control oligodendrocytes (*P =* 0.18), MSA neurons (*P =* 0.16) and control neurons (*P =* 0.18). In contrast, there was a slight decrease in αsyn expression in MSA neurons as compared to control neurons (*P =* 0.46*)*. The greatest difference seems to be the increase in expression in MSA oligodendrocytes compared with MSA neurons. Mann–Whitney U test with significance level set at *P* = 0.05. The boxplot shows median values as the line within the box, the box reflects the interquartile range and the whiskers the range of the values. Oligo: oligodendrocytes; MSA: multiple system atrophy; *SNCA*: α-synuclein.

Next *SNCA* expression in neurons and oligodendrocytes isolated by laser capture was explored in the pontine base and cerebellar white matter, respectively, as these regions are affected in MSA. *SNCA* expression was detected in neurons and oligodendrocytes of both MSA and control cases, with the highest level of expression being found in MSA oligodendrocytes (Fig. 2A). The fold changes in mRNA expression between the different cell types and groups were also calculated (Fig. 2B). Although none of the differences identified reached statistical significance, there was a slight increase in expression in MSA oligodendrocytes as compared to control oligodendrocytes (fold change = 0.7, Mann–Whitney U test, *P =* 0.18). Expression in MSA neurons was slightly decreased as compared to control neurons (fold change = –0.4, Mann–Whitney U test, *P=* 0.46). Comparing the different cell types within the same groups revealed that control oligodendrocytes express 0.3 more *SNCA* than control neurons (Mann–Whitney U test, *P =* 0.92). The greatest difference was seen between MSA oligodendrocytes and neurons where expression in MSA oligodendrocytes was 3.1 times higher than MSA neurons (Mann–Whitney U test, *P =* 0.16).

## Discussion

αSyn has been localized in the brain to neuronal presynaptic terminals and may play a role in neuronal plasticity, vesicular transport, and membrane interaction (Iwai et al. 1995; Kahle et al. 2000; Reynolds et al. 2011). There has been great interest in the possible mechanisms by which αsyn forms primarily oligodendroglial inclusions in MSA since its identification as the main constituent of the GCIs. Understanding the expression pattern of *SNCA* in MSA at both regional and cellular levels is essential to determine if changes in expression play a role in MSA pathogenesis and whether this influences regional vulnerability to disease. The regional expression analysis in this study now demonstrates that *SNCA* expression level varies across different brain regions in control, MSA, and IPD groups. The greatest expression is found in the occipital grey matter and the lowest level in the putamen. However, *SNCA* mRNA expression is not significantly different between the MSA subgroups, IPD and control cases in all regions. The expression trend found in MSA and control cases used in this study is similar to that documented in expression databases such as UKBEC and Allen Brain Atlas, which analyze gene expression in control human brains (Hawrylycz et al. 2012; Trabzuni et al. 2011). The similarity in trend, where *SNCA* is generally expressed more in cortical regions than subcortical and cerebellar regions, has at least two main implications. The first is that the vulnerability of StrN and OPC regions is not associated with a higher baseline expression of *SNCA*. It could be hypothesized that the concentration of GCIs in specific regions is due to higher expression levels of *SNCA* in normal conditions in those regions that is exacerbated by disease. Our data do not support this hypothesis as *SNCA* mRNA levels are higher in cortical than subcortical regions in both normal and disease cases, while GCI pathology is greater in subcortical and hindbrain structures such as the StrN and OPC regions in MSA. This also indicates that the disease does not cause an alteration in expression levels in areas of greater vulnerability. The absence of a difference in *SNCA* expression levels between the different MSA groups, IPD, and controls may imply that factors contributing to pathogenic mechanisms of MSA may be further downstream and not at the transcriptional level and could suggest that impaired αsyn degradation may contribute to the disease. Another possibility is that any differences at the transcriptional level are masked by the cellular heterogeneity of the samples. To overcome this problem, we used LCM to obtain samples highly enriched in neurons and oligodendrocytes.

It has long been suggested that mature oligodendrocytes lack αsyn (Jin et al. 2008; Miller et al. 2005; Solano et al. 2000). However, cell-culture evidence indicates that rat and mouse oligodendrocytes express αsyn protein and mRNA, and in some cases this expression is transient, appearing in precursor cells and declining as they mature (Culvenor et al. 2002; Nielsen et al. 2006; Richter-Landsberg et al. 2000). To date, there is one published study addressing the question of *SNCA* mRNA expression in MSA oligodendrocytes (Miller et al. 2005). This study used double-labeling *in situ* hybridization of *SNCA* and proteolipid protein (PLP), an oligodendrocyte marker, and showed that *SNCA* is not expressed in oligodendrocytes in MSA or control brains. As ISH has limited sensitivity, the findings of this study do not exclude the possibility of *SNCA* mRNA expression by oligodendrocytes (Deglincerti and Jaffrey 2012; Miller et al. 2005).

Analyzing *SNCA* expression in LCM-isolated neurons and oligodendrocytes, our data indicate that this highly conserved protein is expressed in human oligodendrocytes and that expression in MSA oligodendrocytes is greater than control oligodendrocytes. In our hands, LCM isolation of OSP-positive oligodendrocytes provides an oligodendrocyte-enriched sample (Waller et al. 2012). This suggests that *SNCA* overexpression in MSA oligodendrocytes may be a possible mechanism contributing to GCI formation. The cellular expression of αsyn in MSA in this study may also explain the pathological profile of the disease. GCIs are found in greater abundance than NCIs in MSA, and this greater susceptibility of oligodendrocytes to inclusion formation may be a reflection of overexpression of αsyn by oligodendrocytes in MSA.

The novel findings of this LCM study demonstrating expression of *SNCA* mRNA in oligodendrocytes open the door to further exploration of the molecular pathogenesis of MSA. Expanding the current study to examine cellular *SNCA* mRNA expression in other affected and unaffected brain regions in MSA may help to elucidate the selective vulnerability of StrN and OPC regions. In addition to affected and unaffected brain regions, examination of *SNCA* expression in MSA cases with short disease duration and long disease duration may provide further insight into the role of *SNCA* mRNA expression on clinical outcome. A further step would be to characterize *SNCA* mRNA expression in oligodendrocytes with and without GCIs to determine the contribution of *SNCA* expression to inclusion formation in oligodendrocytes. In addition to GCI+/− oligodendrocytes, *SNCA* mRNA expression may also be explored at different stages of oligodendrocyte maturation. The oligodendrocyte lineage comprises precursor, immature, mature nonmyelinating, and mature myelinating cells and αsyn protein in GCIs has been found in mature but not precursor oligodendrocytes in MSA (Ahmed et al. 2013; Papp et al. 1989). Therefore, determining *SNCA* mRNA expression in the different maturational stages may provide further insight into the vulnerability of mature oligodendrocytes to αsyn aggregation.

## References

[b1] Ahmed Z, Asi YT, Lees AJ, Revesz T, Holton JL (2013). Identification and quantification of oligodendrocyte precursor cells in multiple system atrophy, progressive supranuclear palsy and Parkinson's disease. Brain Pathol.

[b2] Ahmed Z, Asi YT, Sailer A, Lees AJ, Houlden H, Revesz T, Holton JL (2012). The neuropathology, pathophysiology and genetics of multiple system atrophy. Neuropathol Appl Neurobiol.

[b3] Beyer K, Domingo-Sàbat M, Humbert J, Carrato C, Ferrer I, Ariza A (2008). Differential expression of alpha-synuclein, parkin, and synphilin-1 isoforms in Lewy body disease. Neurogenetics.

[b4] Culvenor JG, Rietze RL, Bartlett PF, Masters CL, Li Q-X (2002). Oligodendrocytes from neural stem cells express alpha-synuclein: increased numbers from presenilin 1 deficient mice. Neuroreport.

[b5] Deglincerti A, Jaffrey SR (2012). Insights into the roles of local translation from the axonal transcriptome. Open Biol.

[b6] Gwinn-Hardy K, Mehta ND, Farrer M, Maraganore D, Muenter M, Yen SH, Hardy J, Dickson DW (2000). Distinctive neuropathology revealed by alpha-synuclein antibodies in hereditary parkinsonism and dementia linked to chromosome 4p. Acta Neuropathol.

[b7] Hawrylycz MJ, Lein ES, Guillozet-Bongaarts AL, Shen EH, Ng L, Miller JA, van de Lagemaat LN, Smith KA, Ebbert A, Riley ZL, Abajian C, Beckmann CF, Bernard A, Bertagnolli D, Boe AF, Cartagena PM, Chakravarty MM, Chapin M, Chong J, Dalley RA, Daly BD, Dang C, Datta S, Dee N, Dolbeare TA, Faber V, Feng D, Fowler DR, Goldy J, Gregor BW, Haradon Z, Haynor DR, Hohmann JG, Horvath S, Howard RE, Jeromin A, Jochim JM, Kinnunen M, Lau C, Lazarz ET, Lee C, Lemon TA, Li L, Li Y, Morris JA, Overly CC, Parker PD, Parry SE, Reding M, Royall JJ, Schulkin J, Sequeira PA, Slaughterbeck CR, Smith SC, Sodt AJ, Sunkin SM, Swanson BE, Vawter MP, Williams D, Wohnoutka P, Zielke HR, Geschwind DH, Hof PR, Smith SM, Koch C, Grant SGN, Jones AR (2012). An anatomically comprehensive atlas of the adult human brain transcriptome. Nature.

[b8] Iwai A, Masliah E, Yoshimoto M, Ge N, Flanagan L, de Silva HA, Kittel A, Saitoh T (1995). The precursor protein of non-A beta component of Alzheimer's disease amyloid is a presynaptic protein of the central nervous system. Neuron.

[b9] Jellinger KA, Seppi K, Wenning GK (2005). Grading of neuropathology in multiple system atrophy: Proposal for a novel scale. Mov Disord 20(Suppl.

[b10] Jin H, Ishikawa K, Tsunemi T, Ishiguro T, Amino T, Mizusawa H (2008). Analyses of copy number and mRNA expression level of the alpha-synuclein gene in multiple system atrophy. J Med Dent Sci.

[b11] Kahle PJ, Neumann M, Ozmen L, Haass C (2000). Physiology and pathophysiology of alpha-synuclein. Cell culture and transgenic animal models based on a Parkinson's disease-associated protein. Ann N Y Acad Sci.

[b12] Kiely AP, Asi YT, Kara E, Limousin P, Ling H, Lewis P, Proukakis C, Quinn N, Lees AJ, Hardy J, Revesz T, Houlden H, Holton JL (2013). α-Synucleinopathy associated with G51D SNCA mutation: A link between Parkinson's disease and multiple system atrophy?. Acta Neuropathol.

[b13] Kingsbury AE, Daniel SE, Sangha H, Eisen S, Lees AJ, Foster OJF (2004). Alteration in α-synuclein mRNA expression in Parkinson's disease. Mov Disord.

[b14] Langerveld AJ, Mihalko D, DeLong C, Walburn J, Ide CF (2007). Gene expression changes in postmortem tissue from the rostral pons of multiple system atrophy patients. Mov Disord.

[b15] Markopoulou K, Dickson DW, McComb RD, Wszolek ZK, Katechalidou L, Avery L, Stansbury MS, Chase BA (2008). Clinical, neuropathological and genotypic variability in SNCA A53T familial Parkinson's disease. Variability in familial Parkinson's disease. Acta Neuropathol.

[b16] Miller DW, Johnson JM, Solano SM, Hollingsworth ZR, Standaert DG, Young AB (2005). Absence of α-synuclein mRNA expression in normal and multiple system atrophy oligodendroglia. J Neural Transm.

[b17] Nielsen JA, Maric D, Lau P, Barker JL, Hudson LD (2006). Identification of a novel oligodendrocyte cell adhesion protein using gene expression profiling. J Neurosci.

[b18] Obi T, Nishioka K, Ross OA, Terada T, Yamazaki K, Sugiura A, Takanashi M, Mizoguchi K, Mori H, Mizuno Y, Hattori N (2008). Clinicopathologic study of a SNCA gene duplication patient with Parkinson disease and dementia. Neurology.

[b19] Ozawa T, Okuizumi K, Ikeuchi T, Wakabayashi K, Takahashi H, Tsuji S (2001). Analysis of the expression level of alpha-synuclein mRNA using postmortem brain samples from pathologically confirmed cases of multiple system atrophy. Acta Neuropathol.

[b20] Ozawa T, Paviour D, Quinn NP, Josephs KA, Sangha H, Kilford L, Healy DG, Wood NW, Lees AJ, Holton JL, Revesz T (2004). The spectrum of pathological involvement of the striatonigral and olivopontocerebellar systems in multiple system atrophy: Clinicopathological correlations. Brain.

[b21] Papp MI, Kahn JE, Lantos PL (1989). Glial cytoplasmic inclusions in the CNS of patients with multiple system atrophy (striatonigral degeneration, olivopontocerebellar atrophy and Shy-Drager syndrome). J Neurol Sci.

[b22] Reyes JF, Rey NL, Bousset L, Melki R, Brundin P, Angot E (2014). Alpha-synuclein transfers from neurons to oligodendrocytes. Glia.

[b23] Reynolds NP, Soragni A, Rabe M, Verdes D, Liverani E, Handschin S, Riek R, Seeger S (2011). Mechanism of membrane interaction and disruption by α-synuclein. J Am Chem Soc.

[b24] Richter-Landsberg C, Gorath M, Trojanowski JQ, Lee VM (2000). alpha-synuclein is developmentally expressed in cultured rat brain oligodendrocytes. J Neurosci Res.

[b25] Solano SM, Miller DW, Augood SJ, Young AB, Penney JB (2000). Expression of alpha-synuclein, parkin, and ubiquitin carboxy-terminal hydrolase L1 mRNA in human brain: Genes associated with familial Parkinson's disease. Ann Neurol.

[b26] Trabzuni D, Ryten M, Walker R, Smith C, Imran S, Ramasamy A, Weale ME, Hardy J (2011). Quality control parameters on a large dataset of regionally dissected human control brains for whole genome expression studies. J Neurochem.

[b27] Ubhi K, Low P, Masliah E (2011). Multiple system atrophy: A clinical and neuropathological perspective. Trends Neurosci.

[b28] Vandesompele J, De Preter K, Pattyn F, Poppe B, Van Roy N, De Paepe A, Speleman F (2002). Accurate normalization of real-time quantitative RT-PCR data by geometric averaging of multiple internal control genes. Genome Biol.

[b29] Waller R, Woodroofe MN, Francese S, Heath PR, Wharton SB, Ince PG, Sharrack B, Simpson JE (2012). Isolation of enriched glial populations from postmortem human CNS material by immuno-laser capture microdissection. J Neurosci Methods.

